# Profile of small interfering RNAs from cotton plants infected with the polerovirus Cotton leafroll dwarf virus

**DOI:** 10.1186/1471-2199-12-40

**Published:** 2011-08-24

**Authors:** Tatiane F Silva, Elisson AC Romanel, Roberto RS Andrade, Laurent Farinelli, Magne Østerås, Cécile Deluen, Régis L Corrêa, Carlos EG Schrago, Maite FS Vaslin

**Affiliations:** 1 Laboratório de Virologia Molecular Vegetal, Depto. Virologia, IMPPG, Universidade Federal do Rio de Janeiro, Rio de Janeiro, Brasil; 2 Laboratório de Biologia Evolutiva Teórica e Aplicada, Depto. Genética, I. Biologia, Universidade Federal do Rio de Janeiro, Rio de Janeiro, Brasil; 3 Fasteris SA, Plan-les-Ouates, Switzerland; 4 Laboratório de Genômica Funcional e Transdução de Sinal, Depto. Genética, I. Biologia, Universidade Federal do Rio de Janeiro, Rio de Janeiro, Brasil

## Abstract

**Background:**

In response to infection, viral genomes are processed by Dicer-like (DCL) ribonuclease proteins into viral small RNAs (vsRNAs) of discrete sizes. vsRNAs are then used as guides for silencing the viral genome. The profile of vsRNAs produced during the infection process has been extensively studied for some groups of viruses. However, nothing is known about the vsRNAs produced during infections of members of the economically important family *Luteoviridae*, a group of phloem-restricted viruses. Here, we report the characterization of a population of vsRNAs from cotton plants infected with Cotton leafroll dwarf virus (CLRDV), a member of the genus *Polerovirus*, family *Luteoviridae*.

**Results:**

Deep sequencing of small RNAs (sRNAs) from leaves of CLRDV-infected cotton plants revealed that the vsRNAs were 21- to 24-nucleotides (nt) long and that their sequences matched the viral genome, with higher frequencies of matches in the 3- region. There were equivalent amounts of sense and antisense vsRNAs, and the 22-nt class of small RNAs was predominant. During infection, cotton *Dcl *transcripts appeared to be up-regulated, while Dcl2 appeared to be down-regulated.

**Conclusions:**

This is the first report on the profile of sRNAs in a plant infected with a virus from the family *Luteoviridae*. Our sequence data strongly suggest that virus-derived double-stranded RNA functions as one of the main precursors of vsRNAs. Judging by the profiled size classes, all cotton DCLs might be working to silence the virus. The possible causes for the unexpectedly high accumulation of 22-nt vsRNAs are discussed. CLRDV is the causal agent of Cotton blue disease, which occurs worldwide. Our results are an important contribution for understanding the molecular mechanisms involved in this and related diseases.

## Background

The RNA silencing pathway controls important biological processes in plants, including regulation of gene expression during development, heterochromatin formation, hormone signaling, metabolic processes, and stress responses, as well as being an important antiviral defense mechanism [1]. In plants, antiviral silencing can be triggered by the presence of viral double-stranded RNAs (dsRNA), which are generated by the viral RNA polymerase as an intermediate in genomic replication and transcription, or are predicted to form as secondary structures along single stranded viral genomic RNA (ssRNA) [2]. Both structures are recognized by Dicer-like (DCL) ribonucleases and are processed into virus-derived small interfering RNAs (vsRNAs) that vary in length from 21 to 24 nucleotides (nt) [3-5]. These vsRNAs are then loaded into Argonaute (AGO)-containing complexes known as RNA-induced silencing complexes (RISCs), which promote the degradation of both genomic and subgenomic viral RNAs [6,7].

DCL ribonucleases are present in both monocot and dicot plants. *Arabidopsis thaliana *contains four DCLs (AtDCLs1-4) [8], while the *Populus *and rice genomes encode five and six DCLs, respectively [9]. The diversity associated with Dicer ribonucleases, as well as other silencing-related proteins such as AGO, strongly suggest that several silencing pathways have evolved in plants. Correspondingly, in *Arabidopsis*, at least six silencing pathways have been identified, and the four DCLs involved are known to act hierarchically. For example, there are 21-nt vsRNAs and other small RNAs (sRNAs) associated with post-transcriptional silencing of endogenous genes generated by AtDCL4. In the absence of AtDCL4, 22-nt vsRNAs are produced by AtDCL2, and in the absence of both AtDCL4 and AtDCL2, 24-nt vsRNAs are produced by AtDCL3 [10-12]. Thus, AtDCL2-4 play essential roles in mediating the antiviral defenses of *Arabidopsis*. In contrast, AtDCL1 is mainly associated with the production of microRNAs, which represent a class of important regulatory RNAs derived from hairpin-like endogenous transcripts [13].

All of the four Dicer proteins expressed in *Arabidopsis *are usually present in other plants, also [14] (Additional file 1, figure s1). Correspondingly, 21-, 22-, and 24-nt vsRNAs have been detected in many plant hosts following infection [15]. However, based on the hierarchical roles of DCL4 and DCL2 in antiviral silencing, 21-nt vsRNAs are by far the most abundant class of sRNA found in plants infected with RNA or DNA viruses, followed by 22-nt vsRNAs [15-18]. Previous studies have shown that the accumulation of vsRNAs is affected by viral suppressors of gene silencing [11,19].

Suppressor proteins can directly bind vsRNAs [20-23], or inhibit key proteins of the gene-silencing pathway [24-26]. For example, the *Polerovirus *P0 protein and the P38 protein from *Turnip crinkle virus *(TCV) target AGO1, an important antiviral Argonaute protein [11,24,27-30]. Studies have shown that P0 preferentially targets AGO1, leading to its degradation, but does not affect the sRNA-RISC complex [26]. A similar action has been suggested for the P38 protein, which binds to AGO1 and may prevent the assembly of RISC. However, unlike P0, P38 does not affect the stability of AGO1 [30]. By preventing the association of AGO1 with RISC, P38 has the potential to destabilize a complex homeostatic network involving AGO1, microRNAs, and the four Dicer proteins. This would be consistent with the preferential accumulation of 22-nt vsRNAs observed following infection with TCV [30].

In this study, vsRNAs derived from cotton plants (*Gossypium hirsutum*) infected with Cotton leafroll dwarf virus (CLRDV) (genus, *Polerovirus*; family, *Luteoviridae*) were deep-sequenced and characterized. CLRDV is transmitted by the aphid, *Aphis gossypii*, and is the causal agent of cotton blue disease [31], which occurs in cotton crops world-wide. Consistent with other members of the same family, CLRDV is phloem-restricted and its genome consists of a single strand, positive sense, non-polyadenylated RNA (5.8 kb) containing six open reading frames (ORFs) [32]. This is the first report of vsRNAs derived from a member of the family *Luteoviridae *and the first report of vsRNAs in cotton plants.

## Results

### Characterization of CLRDV-derived sRNAs

**Figure 1:**
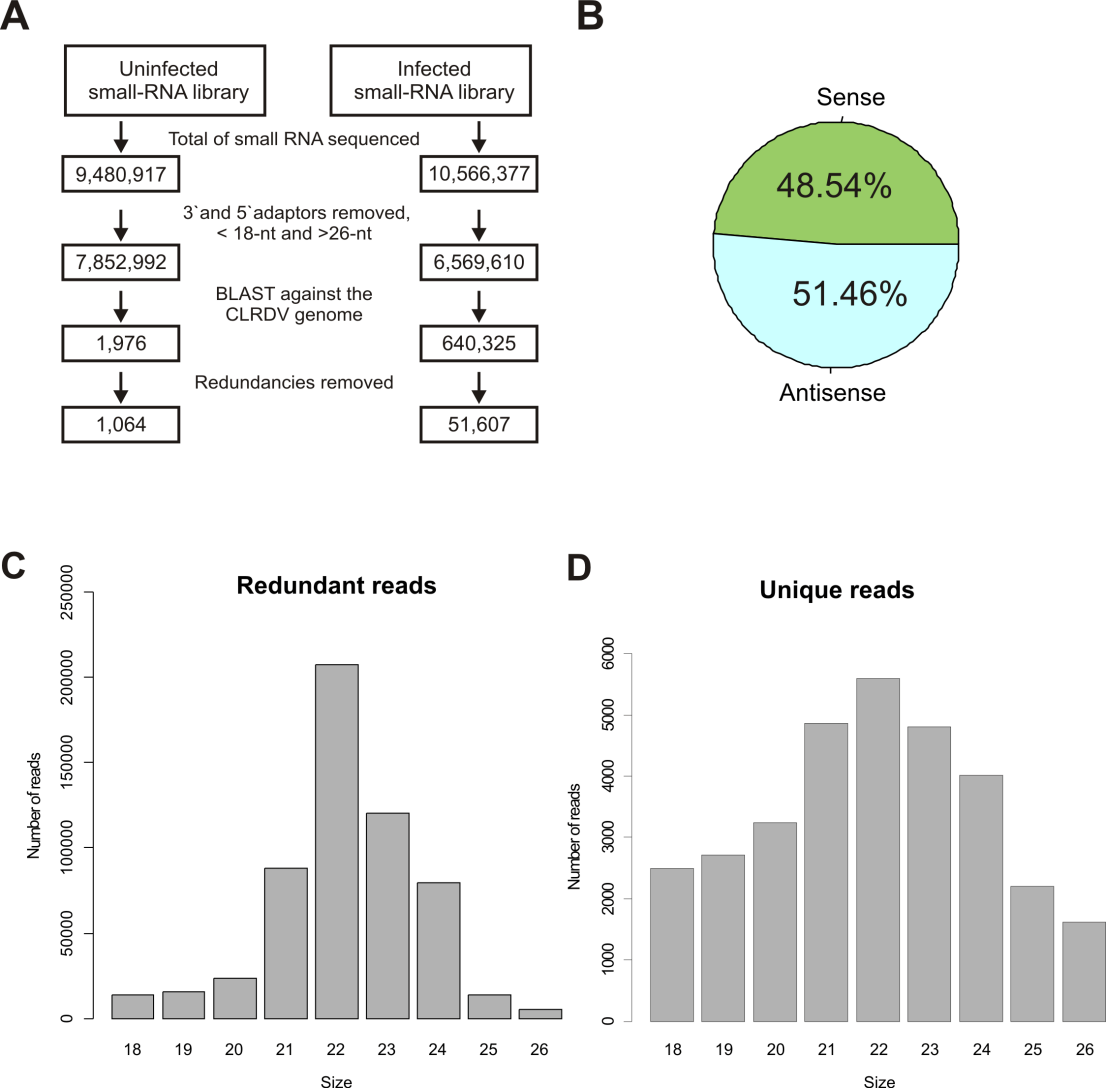
**Analysis of the CLRDV-vsRNA population**. **(A) **Diagram showing the stepwise computational extraction of vsRNA reads from small RNAs libraries recovered from infected and uninfected leaves. (**B**) Accumulation of sense and antisense vsRNAs reads. Percentage for each class of vsRNA from the infected library is shown within the pie graph. (**C**) Histogram representation of total and (**D**) unique vsRNA reads in each size class.

To characterize the vsRNAs produced during CLRDV infection, sRNAs obtained from cotton-infected and uninfected plants were cloned and deep-sequenced using the Illumina platform. A total of 10,566,377 and 9,480,917 reads were sequenced from systemic leaves harvested at 5 dpi from infected and uninfected plants, respectively (Figure 1A). Reads ranging from 18 to 26 nt were mapped in sense and antisense orientations to the viral genome. Only sequences showing no mismatches were regarded as CLRDV vsRNAs in the infected library. In total, 640,325 viral-derived sRNA reads were identified, covering almost the entire sequence of the genome. In the uninfected library, only 1,967 reads matched with the CLRDV genome (corresponding to 0,025% of the 18-26 nt reads sequenced). Of the vsRNA reads identified in infected plants, 51,607 were unique (Figure 1A). Equivalent amounts of sense and antisense vsRNAs were found in the CLRDV-infected cotton library, suggesting that vsRNAs derived from the cleavage of dsRNA are processed by Dicer ribonucleases (Figure 1B). To further characterize the vsRNA library, the frequencies of redundant and unique CLRDV-derived sRNAs ranging from 18 to 26 nt were analyzed (Figure 1). In both sets of analyses, 22-nt vsRNAs were the most abundant. For example, 22-nt redundant vsRNAs (Figure 1C) represented 36.61% of the total vsRNAs sequenced, followed by 23-nt and 21-nt vsRNAs (21.22% and 15.53%, respectively). For the unique vsRNAs (Figure 1D), 22-nt represented 17.74% of the total vsRNAs sequenced, followed by 21-nt and 23-nt vsRNAs (15.42% and 15.23%, respectively). These data were confirmed through the deep sequencing, in an independent channel, of an additional cotton-infected RNA sample (Additional file 2 figure s2), generating 15,415,107 reads, of which 498,367 matched with the CLRDV genome. Together, these results suggest that the cotton homologue of DCL2 (GhDCL2) may be the predominant Dicer ribonuclease involved in their biogenesis.

**Figure 2:**
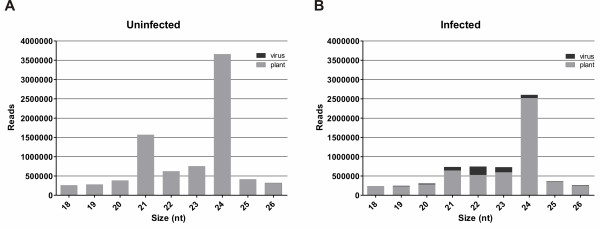
**Size distribution of CLRDV-vsRNA and endogenous sRNAs**. Histograms compare the distribution of 18-26-nt total sRNA reads with vsRNAs obtained from uninfected (**A**) and CLRDV-infected (**B) **cotton plants. Only 0.025% of total sRNA obtained from uninfected plant matched to the CLRDV genome (data not shown).

To determine whether the 22-nt reads are predominant only in the pool of vsRNAs or whether this is the dominant class among small RNAs of cotton, we compared the overall profile of small RNAs between the infected and the uninfected libraries. In both libraries there was an abundance of the 24-nt class among the endogenous sRNAs, followed by the 21-nt class (Figure 2A and 2B). However, in infected plants, there were decreased levels of the 24- and 21-nt classes (Figure 2B) indicating that CLRDV infection may decrease the production of endogenous sRNAs.

The high accumulation of the endogenous 24-nt sRNAs, followed by 21-nt sRNAs, are consistent with sRNA profiles in other plants (33,34). These findings indicate that the cotton RNA silencing machinery responsible for biogenesis of endogenous or viral sRNAs does not tend to produce 22-nt sequences. Therefore, the high levels of 22-nt CLRDV-vsRNA seem to be a result of the antiviral RNA silencing mechanism or a specific CLRDV-host interaction.

**Figure 3:**
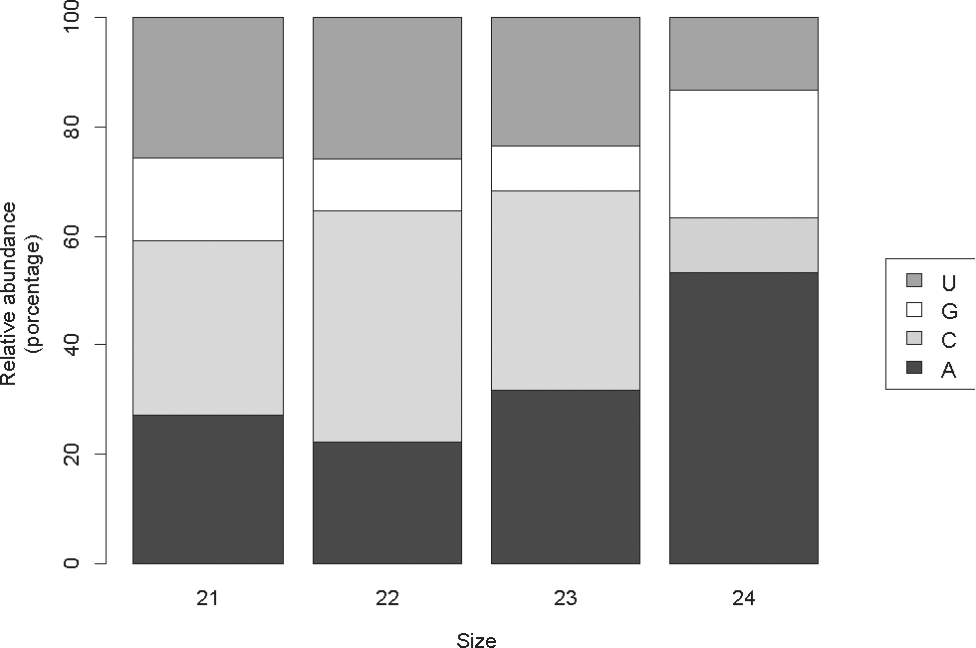
**Characterization of vsRNAs 5' -terminal nucleotide**. Relative abundance of four distinct 5' -terminal nucleotides in 21-24-nt vsRNAs in CLRDV-infected plant.

In *Arabidopsis*, the 5- terminal nucleotide partially determines the preference of sRNAs for AGO proteins. Therefore, the distribution of 5- terminal nucleotides was determined for the sequenced vsRNAs (Figure 3). For all three types of CLRDV-vsRNAs characterized (i.e., 21-, 22- and 23-nt), cytosine was the most commonly occurring nucleotide at the 5- terminus (32.09%, 42.27%, and 36.65%, respectively), while guanine was the least common (14.98%, 9.62%, and 8.12%, respectively). In contrast, 24-nt CLRDV-vsRNAs often had adenine at the 5- terminus (53.24%), or guanine (23.26%). For comparison, *Arabidopsis *AGO1 has a 5- nucleotide preference for uracil, AGO2 and AGO4 have a preference for adenine, and AGO5 preferentially loads sRNAs with cytosine at the 5- terminus [33-35]. Therefore, the results of the present study suggest that CLRDV-vsRNAs can be potentially loaded by multiple AGO-containing complexes. However, 21-23 nt CLRDV-viRNAs may be preferentially loaded by AGO5, while 24-nt CLRDV-vsRNAs would be loaded by AGO 2 and/or AGO4.

### Distribution of vsRNA abundance

**Figure 4:**
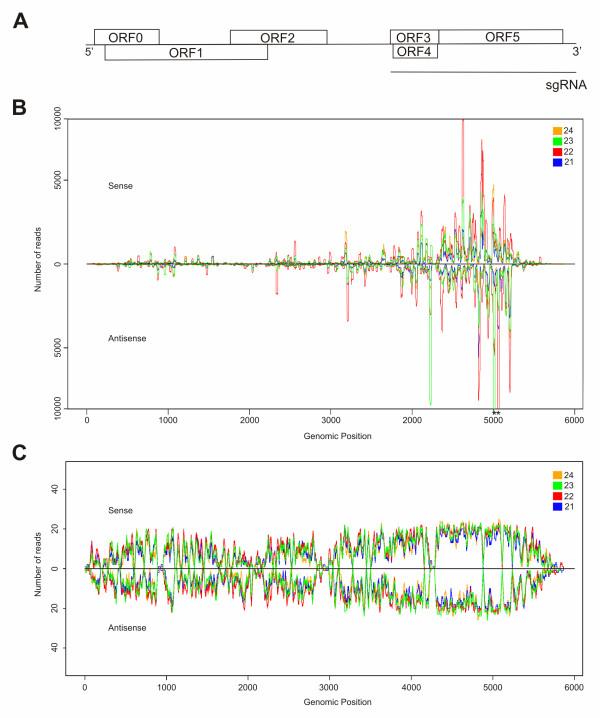
**Genome view of CLRDV-derived sRNAs recovered from infected cotton plants**. (**A**) Schematic representation of CLRDV genome. ORF0 = silencing suppressor protein; ORF1 and ORF2 = virus RNA polymerase; ORF3 = capsid protein; ORF4 = movement protein; ORF3 and ORF5 = readthrough transmission protein. Distribution of total (**B**) and unique (**C**) vsRNAs along viral genome, in either sense (above × axis) or antisense (below × axis) configuration. Figure 4B was scaled to 10000 reads to facilitate the visualization of the obtained data, thus, peaks that eventually were major than 10000 reads were indicated by asterisks.

To examine the spatial distribution and sequence diversity of the viral sRNAs identified, redundant (Figure 4B) and unique (Figure 4C) 21-24 nt vsRNA sequences were mapped to the CLRDV genome. For both classes of sequences, the distribution of CLRDV-vsRNAs along the genome was non-uniform, with most of the vsRNAs accumulating in the 3- region of the genome. This part of the genome encodes structural proteins and proteins that assemble subgenomic RNAs during infection [31]. The greatest numbers of redundant reads were associated with position 5049-5070, with 398,897 reads identified. Overall, this region of the genome was highly represented. In addition, a large number of reads mapped to ORF5, a region encoding an aphid-transmission protein. However, after the unique vsRNAs were aligned with the genome (Figure 4C), there were regions associated with an absence of vsRNA reads. When the same alignment was performed and up to two mismatches were allowed, only positions 4150-4170 did not match with any vsRNA reads (data not shown). Therefore, it is possible that this region may have a structural characteristic that makes it less accessible to Dicer ribonucleases, although sequencing artifacts cannot be ruled out. Furthermore, the hot-spots for vsRNAs that were found to be associated with the 3- region of the CLRDV genome might be generated by the massive accumulation of viral subgenomic RNAs (sgRNA) observed in leaves [15,36].

An analysis of the unique reads that mapped to the CLRDV genome indicated that all Dicer ribonucleases were able to access the entire viral genome. Similar amounts of 21-24-nt vsRNAs corresponding to sense and antisense strands of viral RNA (Figure 4C) were present in our library, reinforcing that virus-derived dsRNAs are the main source of vsRNAs. Furthermore, peaks of both the abundance (Figure 4B) and diversity (Figure 4C) of 21-24 nt reads showed similar patterns of distribution along the genome. These results suggest that all DCL ribonucleases contribute to the generation of vsRNAs with similar substrate affinities and target the same regions of the genome. However, the fact that the 22-nt class of vsRNAs was the most prominent class supports the hypothesis that GhDCL2 may play a role in the generation of CLRDV-vsRNA.

### Expression of Cotton DCL ribonucleases during infection

Assays of TCV infection have detected a high abundance of 22-nt vsRNAs [11,19,30] associated with the silencing suppressor protein, P38. During infection, P38 inactivates AGO1 by down-regulating miR162. As a result, low levels of miR162 directly and/or indirectly affect transcript levels of *Dcl1, 3*, and *4 *[30]. To determine whether a similar mechanism might be activated in cotton-infected plants, we analyzed the expression levels of mature miR162 and *Dcls *in CLRDV-infected and uninfected plants.

**Figure 5:**
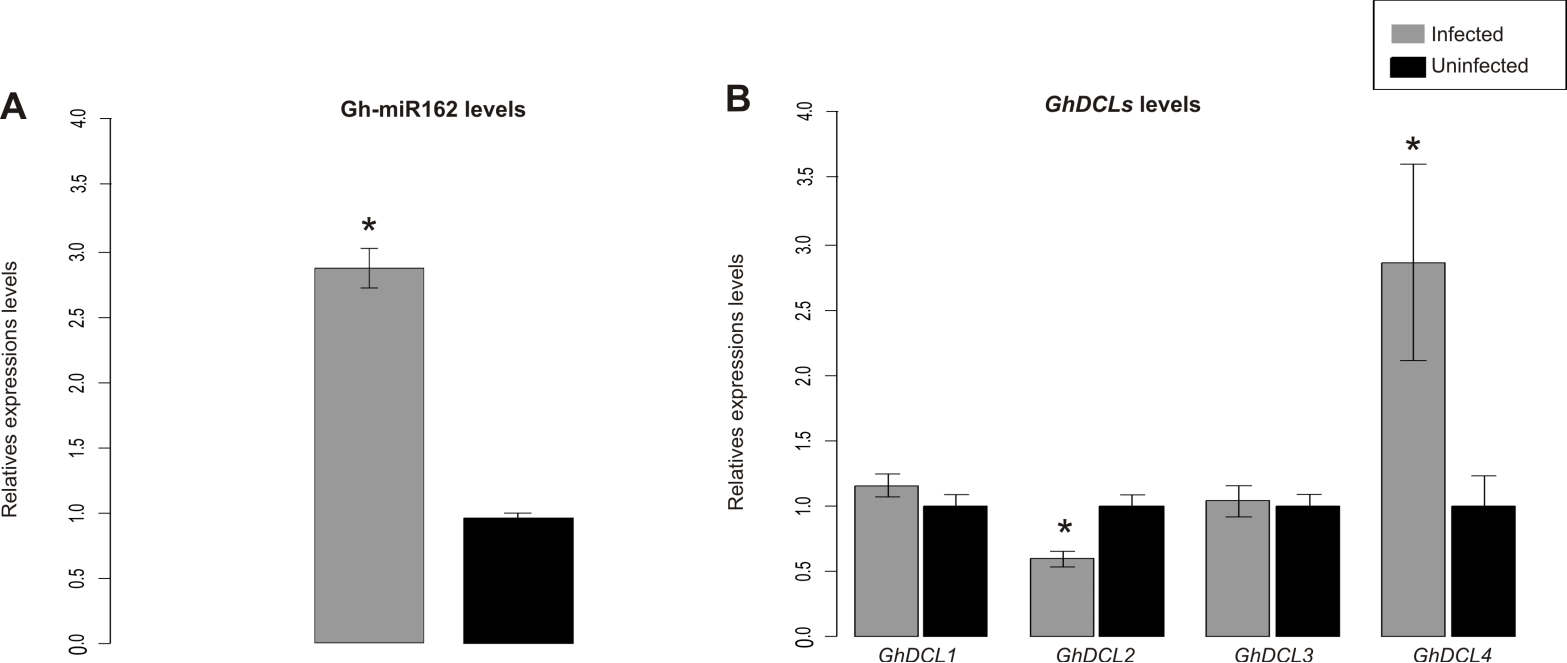
**Quantitative RT-PCR analysis**. Expression levels of Gh-miR162 (**A**) and cotton Dcl mRNAs (**B**) in infected and uninfected plants. Relative expression levels were estimated using polyubiquitin and catalytic subunit of protein phosphatase 2A genes as internal controls. Error bars indicate standard deviations. P values were calculated by t-tests with a significance level of P < 0,05. Asterisks indicate significant differences.

In contrast with TCV infection of *Arabidopsis*, qPCR experiments detected almost three-fold higher levels of Gh-miR162 in infected versus uninfected cotton plants (Figure 5A). Furthermore, *in silico *analysis of miRNAs in the deep-sequencing libraries from infected versus uninfected libraries showed similar results, with Gh-miR162 levels slightly up-regulated during CLRDV infection (data not shown). The levels of mRNAs for cotton DCLs were assayed, and there were no significant differences in the levels of *Dcl1 *(*GhDcl1*) and *Dcl3 *(*GhDcl3*) transcripts (Figure 5B) between uninfected and infected plants. In contrast, *GhDcl4 *was up-regulated during infection, while *GhDcl2 *was down-regulated (Figure 5B). Taken together, these results suggest that the high levels of 22-nt vsRNAs produced in CLRDV-infected plants may represent a mechanism distinct from that previously described for TCV infections.

## Discussion

This is the first report of the characterization of small RNAs produced from a member of the genus *Polerovirus*, family *Luteoviridae*. The profile of vsRNAs generated in cotton plants infected with CLRDV revealed some interesting features regarding their biogenesis. For example, both sense and antisense orientations of CLRDV-derived sRNAs accumulated to similar levels (Figure 1B). However, several other studies have found that sense vsRNAs accumulate to higher levels in some hosts [16,17,23]. In those cases, strand biases are usually attributed to preferential processing of highly structured single-stranded genomic viral RNAs by Dicer ribonucleases [15,23]. Despite these differences and considerable experimental efforts, however, the existence of a direct correlation between vsRNA hot-spots and structured regions of genomic viral RNAs has never been proven [15]. The accumulation of equivalent amounts of sense and antisense CLRDV-vsRNAs observed in the present study supports the hypothesis that CLRDV-dsRNAs, which are generated by viral RNA polymerases during genome replication or by the activity of host RNA-dependent RNA polymerases [2], are the main substrates for Dicer ribonucleases. Since the P0 silencing suppressor protein from *Polerovirus *was already shown to inhibit production of secondary vsRNAs in 35S-promoter-driven agroinfiltration assays [27,28,37], it may be speculated that the main substrate of cotton DCLs during CLRDV infection is probably the replicative intermediate forms of viral genomic RNAs. However, the mechanism of P0 protein action in the formation of the secondary siRNAs during virus infection remains unclear.

Overall, the distribution profile of CLRDV-vsRNAs within the genome varied considerably. High vsRNA densities were identified in regions coding for structural proteins, especially in the ORF5 region (Figure 4). Previously, it was shown that genes encoding structural proteins in the family *Luteoviridae *are expressed from subgenomic RNAs (i.e., sgRNA1 and sgRNA2) [38]. Moreover, studies of the *Polerovirus*, Potato leafroll virus (PLRV), also identified two sgRNAs associated with the 3- block of the viral genome [39]. The transcription of sgRNA1 provides for expression of ORFs 3, 4, and 5, while that of sgRNA2 (~800 nt) encodes two proteins located within the 3- proximal half of ORF5. Since sgRNAs are highly expressed during the infection cycle, an over-accumulation of vsRNAs derived from this region of the genome might be due to a greater availability of dsRNA intermediate templates for processing. Accordingly, the hot-spot of vsRNAs mapped to ORF5 might be due to the expression of sgRNA2, which is also derived from this region of the genome. Although the synthesis of sgRNA2 by CLRDV has not previously been reported, the ACAAAA motif present at the 5- end of sgRNA1 and sgRNA2 from other Poleroviruses [40] is also present in the ORF5 of CLRDV (position 4821-4828) (data not shown). Based on these results, it is possible that sgRNA2 is produced by CLRDV.

Depending on their length and 5- identity, sRNAs are selectively loaded into multiple AGO complexes [33,34]. Previous studies have shown that plant virus-specific sRNAs beginning with uracil or adenine are preferentially loaded into AGO1, AGO2, and AGO4 [15-17]. In fact, AGO1 and AGO2 are required for the anti-viral silencing pathway in *Arabidopsis *[41-43]. However, 21-23 nt CLRDV-vsRNAs usually have a cytosine at the 5- terminal position (Figure 3), indicating that they may be loaded into a cotton homologue of AGO5. Although the AtAGO5 has no detectable anti-viral function against Cucumber mosaic virus (CMV) [8,43,44], CMV-vsRNAs have been detected in AtAGO5 immunoprecipitates, indicating that the protein may act in the biogenesis of secondary vsRNAs [35]. Moreover, a predominance of 5- terminal cytosines has been observed for some viroid-derived sRNAs [45]. In contrast, most 24-nt CLRDV-vsRNAs have adenine at the 5- terminal (Figure 3), indicating that they can be loaded into cotton AGO2 and AGO4 homologues. In *Arabidopsis*, the association between 24-nt sRNAs and AGO4 has been well-characterized as a mediator of transcriptional silencing for transposons and repeated sequences [41]. In addition, the decreased number of vsRNAs that start with guanine is correlated with the absence of AGO proteins that might otherwise have an affinity for those sRNAs.

The balance between antiviral silencing and suppression mechanisms can directly influence the accumulation of vsRNAs within infected plants. While the functions of the four DCL proteins present in *Arabidopsis *are well characterized, Dicer ribonucleases from other species, including cotton, remain largely unstudied. However, if the mechanism(s) associated with DCL ribonucleases is conserved between cotton and *Arabidopsis*, then the predominance of 22-nt vsRNAs associated with CLRDV infection would be hypothesized to be the result of GhDCL2 activity. Although 22-nt CMV-vsRNAs produced by AtDCL2 are poor effectors of antiviral defense in *Arabidopsis *[43], other studies have detected a predominant population of 22-nt vsRNAs following infection with certain plant viruses and viroids [11,15,19,36,45]. For example, Cymbidium ring spot virus (CymRSV) and TCV infections are associated with an abundance of 22-nt vsRNAs, which seem to be related to the activity of the suppressor proteins P19 and P38 [11,15,19]. P19 can specifically sequester 21-nt duplex sRNAs [46], while P38 can indirectly block AtDCL4 activity by suppressing AGO1 function [30]. During TCV infection in *Arabidopsis, AtDCL1 *levels are indirectly increased due to the P38-mediated down-regulation of microRNAs, including miR162, a negative regulator of *AtDcl1 *transcripts [47]. Since AtDCL1 negatively regulates *AtDcl4 *and *AtDcl3 *[48], over-accumulation of AtDCL1 generates a deficit in the levels of AtDCL4 and AtDCL3, leaving dsRNAs more accessible to AtDCL2 [30]. The *Polerovirus *P0 suppressor protein is also able to destabilize AGO1 [24,27,28]. Although the activity of CLRDV P0 has not yet been tested, the F-Box-like domain necessary for silencing that is conserved among P0 sequences from other members of the genus is also conserved in CLRDV (data not shown). Thus, CLRDV P0 has the potential to similarly affect cotton Dicer ribonucleases during the infection process. However, in this study, there were no significant changes in the levels of *GhDcl1, GhDcl2*, and *GhDcl3 *transcripts in infected plants (Figure 5B). Furthermore, *GhDcl4 *transcripts and Gh-miR162 were up-regulated (Figure 5A and 5B). The up-regulation of *Dcl4 *has been observed in other viral infections [49], but the levels of mature miR162 are inconsistent with what was observed during TCV infection [30]. It is possible that differences in tissue tropism between TCV and CLRDV, and/or differences in the silencing machinery of the host, account for the observed differences between the two viruses.

Members of the genus *Polerovirus *are restricted to the phloem cells of their hosts. Therefore, DCL activity in response to viral dsRNA may be cell-type dependent. Small RNAs derived from Hop stunt viroid (HSVd) infections in cucumber plants showed different sizes in different tissues [45]. For example, most of the sRNAs from infected whole leaves were 21-nt long, while those derived from phloem-sap were more frequently 22 nt in length. Although transgene-induced silencing in phloem cells of *Arabidopsis *is triggered by AtDCL4 [50], a difference in the affinity or expression of Dicer ribonucleases, or other silencing-related proteins such as dsRNA-binding proteins in companion cells, could possibly explain the tissue-dependent shift in sRNA size.

The production of vsRNAs following virus infection can vary depending on the host. For example, sRNAs derived from Bamboo mosaic virus are mainly 21 nt in length in *Arabidopsis*, but 22 nt in *Nicotiana benthamiana*. Therefore, these data suggest that DCL recruitment for vsRNA production is a host-dependent process [36]. This is the first report of a sRNA profile for cotton virus-infected plants. Further research is required to confirm whether the vsRNA profile observed here results from a viral silencing suppressor protein, or from factors such as phloem-restriction or cotton-specific factors that can activate an anti-viral silencing pathway.

## Conclusions

This is the first high-throughput sequencing of a member of the *Luteoviridae *family, CLRDV, from virus-infected cotton plants. This study shows that RNA silencing systems against CLRDV result in the production of 22-nt sRNAs as the predominant sRNA size class. All vsRNAs, independently of the size, and that these are derived mainly from the 3- region of the viral genome. The sequence data of sense and antisense vsRNAs strongly suggest that dsRNA molecules are the main source of the vsRNAs. During CLRDV infection, we observed up-regulation of *GhDcl4 *and down-regulation of *GhDcl2 *transcripts, which are the major DCLs in antiviral defense in the model plant *Arabidopsis*. There is still much to learn about the molecular mechanisms underlying the prevalence of the 22-nt CLRDV-vsRNAs.

## Methods

### Sample preparation and sequencing

Fifty-day-old cotton (*Gossypium hirsutum*) plants (cultivar FM966; Fibermax966) that are susceptible to cotton blue disease were infected with CLRDV using the viruliferous aphid, *Aphis gossypii*. Aphids were placed on older true leaves and removed 24 h after infestation. Systemic leaves (i.e., representing the youngest completely expanded leaves) were harvested 5 days post-infection (dpi). The same leaves were harvested from mock-infected plants as the control. Total RNAs were extracted from systemic leaves using the Invisorb Spin Plant RNA Mini Kit (Invisorb^®^).

The quantity and quality of RNA samples obtained were determined by spectrophotometry (Nanodrop ND-1000, Thermo Fisher Scientific) and agarose gel electrophoresis (Additional file 3, figure s3), respectively. Systemic infections were confirmed using nested (RT)-PCR assays to detect the viral capsid protein-encoding gene as previously described [51]. RNA samples were precipitated in ethanol and sequenced at the Fasteris Life Science Co. (Geneva, Switzerland) with an Illumina Genome Analyzer (Illumina, San Diego, USA). Small RNA libraries were prepared according to a modified Illumina protocol. Briefly, small RNAs of 15-30 nt were purified on an acrylamide gel; the 3- IDT miRNA cloning linker (Integrated DNA Technologies, San Diego, USA) and then the 5- Illumina adapters were single-stranded ligated with T4 RNA ligase. The constructs were purified again on an acrylamide gel to remove empty adapters and then reverse-transcribed and PCR-amplified. The primers used for cDNA synthesis and PCR were designed to insert an index in the 3- adapter. The libraries were quality controlled by cloning an aliquot into a TOPO plasmid and capillary sequencing 4-8 clones. High-throughput sequencing was performed on a Genome Analyzer GAIIx for 38 cycles plus 7 cycles to read the indexes. After demultiplexing and adapter removal, 10.5 million pass filter reads were obtained in the library.

All the deep sequencing libraries obtained are deposited at GEO (Gene Expression Omnibus) under the number GSE311062 http://www.ncbi.nlm.nih.gov/geo/info/submission.html

### Data mining of the sRNA pool

CLRDV-derived sRNAs sequences were identified using a local BLAST database of the CLRDV-PV1 isolate genomic sequence (accession number HQ827780). Library characterization and mapping to the viral genome were performed using locally developed Perl scripts. Further calculations and statistical analyses were performed using R 2.7.1 software (R Foundation for Statistical Computing).

### Real-time analyses

Primers used to amply the DCLs genes and Gh-miR162 are listed in Table 1.

**Table 1:** Primer sequences and amplicon characteristics of DCLs, XTH, and Gh-miR162

Gene	Forward primer sequence (5'-3')	Reverse primer sequence (5'-3')	Amplicon size (bp)	Efficiency ± SD*	R2	Locus accession number
DCL1	AACCCTGGGTGGTGTCCCCTG	ATGCCCCCTTTTGGCTGGCTC	132	0.928647 ± 0.0293724	0.9858	ES804646.1
DCL2	GATCGCTATCATGCTTCTCCGCAG	TGGGGAACCAAGAAGACAGCGAA	81	0.99522 ± 0.00361129	0.9903	DW488144
DCL3	ATGTCCACATGCCCCCTGAGCT	GGCCAACATTAAGGACTCCAGCCG	113	0.997162 ± 0.0071986	0.9646	DR462994
DCL4	GCTTCCAAGCGGCAACAGCATT	AGGATGCACAATCGCCTGAAGGAG	186	0.992603 ± 0.0051324	0.9706	DT568872
Gh-miR162	GCGGCGGAGCTATTTGGAGACG	GTTGGCTCTGGTGCAGGGTCCGAGGTATTCGCACCAGAGCCAACCTGGAT	72	0.884982 ± 0.032819	0.8519	-
XTH	GGAAAGGGTGACAGGGAACA	GGCTGGAGTTTTGGGTATGG	173 and 392**	-	-	AY189971.2

*Efficiency ± standard deviation (SD) generated by the Miners software. ** in DNA sample.

To measure expression levels of mature Gh-miR162, a stem-loop quantitative RT-PCR technique was used as previously described [52].

Complementary DNA was produced using the RevertAid First Strand cDNA Synthesis Kit (Fermentas) and 0.5 μg of total RNA previously treated with DNase I (Fermentas). cDNAs of the cotton DCL genes were synthesized by adding 100 μM Oligo (dT24V) primer. For synthesis of Gh-miR162 cDNA, 100 μM specific primer was added (Table 1). The presence of residual genomic DNA in the RNA samples was verified by PCR of the control gene xyloglucan endotransglycosylase (XTH) (accession number AY189971.2), using primers spanning two exons and RNA samples that were not reverse-transcribed (RT) (Additional file 4, figure s4).

Synthesized cDNAs were diluted 50 times and 2.5 μL of these dilutions were analyzed by quantitative PCR (qPCR). Assays were performed using a 48-well plate on an Step One Real-Time PCR system (Applied Biosystems) with Maxima™ SYBR Green/ROX qPCR Master Mix (Fermentas), following the manufacturer's instructions. The cycling conditions were as follows: 10 min at 95°C for initial denaturation, followed by 40 cycles of denaturation at 95°C for 15 s and annealing/extension at 60°C for 30 s. Results were normalized against cotton genes for polyubiquitin (accession number DW505546) and the catalytic subunit of phosphatase 2A (accession number DT545658) [53]. The reference genes were validated experimentally in specific CLRDV-infected samples (Additional file 5, figure s5). All reactions were performed using two independent biological samples and each sample was analyzed in triplicate wells. The mean value of each Ct triplicate was used for further calculations by the 2^-ΔCt ^method. Each PCR run included a no-template control containing water instead of cDNA.

The efficiency values of the DCLs and Gh-miR162 primers sets were estimated for each experimental set by Miner software [54], and are listed in Table 1. Amplification of a specific transcript was confirmed by the appearance of a single peak in the melting curve followed by agarose gel electrophoresis (Additional file 6, figure s6). The correlation coefficient (R2) was calculated for each transcript (Table 1). The values shown are averages obtained from three biological replicates, and relative expression levels were obtained by comparing infected plants with uninfected plants.

## Authors' contributions

TFS, RLC, EAdCR, and MSFV conceived and designed the experiments. TFS, LF, MØ, and CD performed the experiments. TFS, RRSA, EAdCR, and CEGS carried out bioinformatics analyses and analyzed the data. TFS, RLC, LF and MSFV contributed reagents, materials and analytical tools. TFS, RLC and MSFV wrote the paper. All authors read and approved the final manuscript.

## Supplementary Material

Additional file 1 **Phylogenetic relationship between cotton Dicer ribonucleases and their homologues in other species**. **A**, **B**, **C**, and **D**, Unrooted Neighbor-joining tree constructed with DCL1, DCL2, DCL3, or DCL4 homologue sequences, respectively. Species used in the phylogeny were as follows: *Arabidopsis thaliana *(At), *Gossypium hirsutum *(Gh), *Medicago truncatula *(Mt), *Oryza sativa *(Os), *Physcomitrella patens *(Pp), *Populus trichocarpa *(Pt) and *Vitis vinifera *(Vv). Dashes below each tree represent amino acid regions used in alignment. Arrows represent fragments analyzed by qPCR. Bootstrap values from 1,000 replicates were used to assess the robustness of the trees. All DCL sequences, except cotton DCLs, were downloaded from Phytozome 6.0 http://www.phytozome.net/. ESTs from *G. hirsutum *containing incomplete DCL sequences were obtained from the NCBI database. The GhDCL1 consensus sequence was constructed with ESTs DT564382.1 (Helicase domain), and ES804646.1, together with DW238156.1 (two RNAse III and one Double stranded RNA binding (dsRB) domain). The GhDCL2 consensus sequence was constructed from two ESTs: DW484144 (DEAD-like helicases superfamily (DExD) domain) and ES806737 (second RNAse III domain). The GhDCL3 sequence was constructed from the ESTs DW477937 and DR462994 (PAZ and RNAse III domains, respectively). The GhDCL4 consensus sequence was constructed with ESTs ES841096 (PAZ domain) and DT568872 (RNAse III domain). Smart database [55] was used to identity DCL domains from their amino acid sequences. Click here for file

Additional file 2 **Analysis of biological duplicates of CLRDV-vsRNA populations**. Histogram showing total (**A**) and unique (**B**) vsRNA reads in each size class. Biological duplicates were subjected to deep sequencing in independent channels. Click here for file

Additional file 3 **Total RNA quality check**. Quality and integrity of each RNA sample was checked by electrophoresis on 0,8% non-denaturing agarose gels, as well as by absorbance at 260 and 280 nm. Click here for file

Additional file 4 **Confirmation of DNA-free status of RNA samples**. DNA contamination was checked by 2.0% agarose gel electrophoresis of products obtained in the xyloglucan endotransglycosylase (XTH) gene amplification reaction. Different sized fragments are amplified from genomic DNA (392 bp) and mRNA transcripts from cDNA (173 bp) with the designed primers. Before reverse transcription (-RT) reactions, RNA samples were used for PCR reactions and showed no amplification. Infected; RNA samples from plants independently infected with CLRDV. Uninfected; two biologically independent RNA samples from uninfected plants. DNA; genomic DNA amplification (positive control). Click here for file

Additional file 5 **Determination of reference genes for use in these experimental conditions**. Expression stability values of polyubiquitin (UBI), the catalytic subunit of phosphatase 2A (PP2A), and 18S ribosomal RNA (18S) candidate reference genes obtained by different algorithms. (**A**) Normfinder. (**B**) Delta CT method. (**C**) BestKeeper. (**D**) Genenorm. In Gennorm analysis, 0.15 is the cut-off value below which the inclusion of an additional reference gene is not required [56]. All analyses were performed via the Cotton EST Database http://www.leonxie.com/index.php. Click here for file

Additional file 6 **Test of specificity of RT-qPCR primers**. (**A) **Melting curves of the four *GhDcls *and Gh-miR162 sequence-related RNAs after RT-qPCR using SYBR-green. (**B**) Non-denaturing agarose (2.0%) gel electrophoresis showing amplification of single products with the expected size for each of the GhDCL gene transcripts and Gh-miR162. M represents O'GeneRuler 100 bp DNA Ladder (Fermentas). Click here for file

## References

[B1] Voinnet O Origin, biogenesis, and activity of plant microRNAs Cell 2009 136 4 669 687 10.1016/j.cell.2009.01.046 19239888

[B2] Llave C Virus-derived small interfering RNAs at the core of plant-virus interactions Trends in Plant Science 2010 15 12 701 707 10.1016/j.tplants.2010.09.001 20926332

[B3] Hamilton AJ Baulcombe DC A species of small antisense RNA in posttranscriptional gene silencing in plants Science 1999 286 5441 950 952 10.1126/science.286.5441.950 10542148

[B4] Zamore PD Tuschl T Sharp PA Bartel DP RNAi: double-stranded RNA directs the ATP-dependent cleavage of mRNA at 21 to 23 nucleotide intervals Cell 2000 101 1 25 33 10.1016/S0092-8674(00)80620-0 10778853

[B5] Zamore PD Haley B Ribo-gnome: the big world of small RNAs Science 2005 309 5740 1519 1524 10.1126/science.1111444 16141061

[B6] Fagard M Boutet S Morel JB Bellini C Vaucheret H AGO1, QDE-2, and RDE-1 are related proteins required for post-transcriptional gene silencing in plants, quelling in fungi, and RNA interference in animals Proc Natl Acad Sci USA 2000 97 21 11650 11654 11016954 10.1073/pnas.200217597 PMC17255

[B7] Baumberger N Baulcombe DC Arabidopsis ARGONAUTE1 is an RNA Slicer that selectively recruits microRNAs and short interfering RNAs Proc Natl Acad Sci USA 2005 102 33 11928 11933 10.1073/pnas.0505461102 16081530 PMC1182554

[B8] Brodersen P Voinnet O The diversity of RNA silencing pathways in plants Trends in Genetics 2006 22 5 268 280 10.1016/j.tig.2006.03.003 16567016

[B9] Margis R Fusaro AF Smith NA Curtin SJ Watson JM Finnegan EJ Waterhouse PM The evolution and diversification of Dicers in plants FEBS Letters 2006 580 10 2442 2450 10.1016/j.febslet.2006.03.072 16638569

[B10] Bouche N Lauressergues D Gasciolli V Vaucheret H An antagonistic function for Arabidopsis DCL2 in development and a new function for DCL4 in generating viral siRNAs EMBO Journal 2006 25 14 3347 3356 10.1038/sj.emboj.7601217 16810317 PMC1523179

[B11] Deleris A Gallego-Bartolome J Bao J Kasschau KD Carrington JC Voinnet O Hierarchical action and inhibition of plant Dicer-like proteins in antiviral defense Science 2006 313 5783 68 71 10.1126/science.1128214 16741077

[B12] Fusaro AF Matthew L Smith NA Curtin SJ Dedic-Hagan J Ellacott GA Watson JM Wang MB Brosnan C Carroll BJ RNA interference-inducing hairpin RNAs in plants act through the viral defence pathway EMBO Reports 2006 7 11 1168 1175 10.1038/sj.embor.7400837 17039251 PMC1679793

[B13] Liu B Li PC Li X Liu CY Cao SY Chu CC Cao XF Loss of function of OsDCL1 affects microRNA accumulation and causes developmental defects in rice Plant Physiology 2005 139 1 296 305 10.1104/pp.105.063420 16126864 PMC1203379

[B14] Liu QP Feng Y Zhu ZJ Dicer-like (DCL) proteins in plants Functional & Integrative Genomics 2009 9 3 277 286 10.1007/s10142-009-0111-5 19221817

[B15] Donaire L Wang Y Gonzalez-Ibeas D Mayer KF Aranda MA Llave C Deep-sequencing of plant viral small RNAs reveals effective and widespread targeting of viral genomes Virology 2009 392 2 203 214 10.1016/j.virol.2009.07.005 19665162

[B16] Donaire L Barajas D Martinez-Garcia B Martinez-Priego L Pagan I Llave C Structural and genetic requirements for the biogenesis of tobacco rattle virus-derived small interfering RNAs Journal of Virology 2008 82 11 5167 5177 10.1128/JVI.00272-08 18353962 PMC2395200

[B17] Qi X Bao FS Xie Z Small RNA deep sequencing reveals role for Arabidopsis thaliana RNA-dependent RNA polymerases in viral siRNA biogenesis PLoS ONE 2009 4 3 e4971 10.1371/journal.pone.0004971 19308254 PMC2654919

[B18] Yan F Zhang HM Adams MJ Yang J Peng JJ Antoniw JF Zhou YJ Chen JP Characterization of siRNAs derived from rice stripe virus in infected rice plants by deep sequencing Archives of Virology 2010 155 6 935 940 10.1007/s00705-010-0670-8 20396917

[B19] Xie Z Johansen LK Gustafson AM Kasschau KD Lellis AD Zilberman D Jacobsen SE Carrington JC Genetic and functional diversification of small RNA pathways in plants PLoS Biology 2004 2 5 E104 10.1371/journal.pbio.0020104 15024409 PMC350667

[B20] Lakatos L Csorba T Pantaleo V Chapman EJ Carrington JC Liu YP Dolja VV Calvino LF Lopez-Moya JJ Burgyan J Small RNA binding is a common strategy to suppress RNA silencing by several viral suppressors EMBO Journal 2006 25 12 2768 2780 10.1038/sj.emboj.7601164 16724105 PMC1500863

[B21] Merai Z Kerenyi Z Kertesz S Magna M Lakatos L Silhavy D Double-stranded RNA binding may be a general plant RNA viral strategy to suppress RNA silencing Journal of Virology 2006 80 12 5747 5756 10.1128/JVI.01963-05 16731914 PMC1472586

[B22] Csorba T Bovi A Dalmay T Burgyan J The p122 subunit of Tobacco Mosaic Virus replicase is a potent silencing suppressor and compromises both small interfering RNA- and microRNA-mediated pathways Journal of Virology 2007 81 21 11768 11780 10.1128/JVI.01230-07 17715232 PMC2168790

[B23] Ding SW Voinnet O Antiviral immunity directed by small RNAs Cell 2007 130 3 413 426 10.1016/j.cell.2007.07.039 17693253 PMC2703654

[B24] Pazhouhandeh M Dieterle M Marrocco K Lechner E Berry B Brault V Hemmer O Kretsch T Richards KE Genschik P F-box-like domain in the polerovirus protein P0 is required for silencing suppressor function Proc Natl Acad Sci USA 2006 103 6 1994 1999 10.1073/pnas.0510784103 16446454 PMC1413668

[B25] Zhang X Jacobsen SE Genetic analyses of DNA methyltransferases in Arabidopsis thaliana Cold Spring Harb Symp Quant Biol 2006 71 439 447 10.1101/sqb.2006.71.047 17381326

[B26] Csorba T Lozsa R Hutvagner G Burgyan J Polerovirus protein P0 prevents the assembly of small RNA-containing RISC complexes and leads to degradation of ARGONAUTE1 The Plant Journal 62 463 472 20128884 10.1111/j.1365-313X.2010.04163.x

[B27] Bortolamiol D Pazhouhandeh M Marrocco K Genschik P Ziegler-Graff V The Polerovirus F box protein P0 targets ARGONAUTE1 to suppress RNA silencing Current Biology 2007 17 18 1615 1621 10.1016/j.cub.2007.07.061 17869109

[B28] Baumberger N Tsai CH Lie M Havecker E Baulcombe DC The Polerovirus silencing suppressor P0 targets ARGONAUTE proteins for degradation Current Biology 2007 17 18 1609 1614 10.1016/j.cub.2007.08.039 17869110

[B29] Pfeffer S Dunoyer P Heim F Richards KE Jonard G Ziegler-Graff V P0 of beet Western yellows virus is a suppressor of posttranscriptional gene silencing Journal of Virology 2002 76 13 6815 6824 10.1128/JVI.76.13.6815-6824.2002 12050394 PMC136274

[B30] Azevedo J Garcia D Pontier D Ohnesorge S Yu A Garcia S Braun L Bergdoll M Hakimi MA Lagrange T Argonaute quenching and global changes in Dicer homeostasis caused by a pathogen-encoded GW repeat protein Genes & Development 2010 24 9 904 915 10.1101/gad.1908710 20439431 PMC2861190

[B31] Correa RL Silva TF Simoes-Araujo JL Barroso PA Vidal MS Vaslin MF Molecular characterization of a virus from the family Luteoviridae associated with cotton blue disease Archives of Virology 2005 150 7 1357 1367 10.1007/s00705-004-0475-8 15789270

[B32] Distéfano AJ Bonacic Kresic I Hopp HE The complete genome sequence of a virus associated with cotton blue disease, cotton leafroll dwarf virus, confirms that it is a new member of the genus Polerovirus Archives of Virology 2010 155 1849 1854 10.1007/s00705-010-0764-3 20677026

[B33] Mi S Cai T Hu Y Chen Y Hodges E Ni F Wu L Li S Zhou H Long C Sorting of small RNAs into Arabidopsis argonaute complexes is directed by the 5' terminal nucleotide Cell 2008 133 1 116 127 10.1016/j.cell.2008.02.034 18342361 PMC2981139

[B34] Montgomery TA Howell MD Cuperus JT Li D Hansen JE Alexander AL Chapman EJ Fahlgren N Allen E Carrington JC Specificity of ARGONAUTE7-miR390 interaction and dual functionality in TAS3 trans-acting siRNA formation Cell 2008 133 1 128 141 10.1016/j.cell.2008.02.033 18342362

[B35] Takeda A Iwasaki S Watanabe T Utsumi M Watanabe Y The mechanism selecting the guide strand from small RNA duplexes is different among argonaute proteins Plant Cell Physiology 2008 49 4 493 500 10.1093/pcp/pcn043 18344228

[B36] Lin KY Cheng CP Chang BCH Wang WC Huang YW Lee YS Huang HD Hsu YH Lin NS Global Analyses of Small Interfering RNAs Derived from Bamboo mosaic virus and Its Associated Satellite RNAs in Different Plants PLoS ONE 2010 5 8 e11928 10.1371/journal.pone.0011928 20689857 PMC2914070

[B37] Mangwende T Wang ML Borth W Hu J Moore PH Mirkov TE Albert HH The P0 gene of Sugarcane yellow leaf virus encodes an RNA silencing suppressor with unique activities Virology 2009 384 1 38 50 10.1016/j.virol.2008.10.034 19046592

[B38] Kelly L Gerlach WL Waterhouse PM Characterisation of the subgenomic RNAs of an Australian isolate of barley yellow dwarf luteovirus Virology 1994 202 2 565 573 10.1006/viro.1994.1378 8030222

[B39] Ashoub A Rohde W Prufer D In planta transcription of a second subgenomic RNA increases the complexity of the subgroup 2 luteovirus genome Nucleic Acids Research 1998 26 2 420 426 10.1093/nar/26.2.420 9421494 PMC147298

[B40] Mayo MA Ziegler-Graff V Molecular biology of luteoviruses Advances in Virus Research 1996 46 413 460 8824705 10.1016/s0065-3527(08)60077-9

[B41] Chitwood DH Timmermans MCP Small RNAs are on the move Nature 2010 467 7314 415 419 10.1038/nature09351 20864994

[B42] Harvey JJW Lewsey MG Patel K Westwood J Heimstadt S Carr JP Baulcombe DC An Antiviral Defense Role of AGO2 in Plants PLoS ONE 2011 6 1 e14639 10.1371/journal.pone.0014639 21305057 PMC3031535

[B43] Wang XB Jovel J Udomporn P Wang Y Wu Q Li WX Gasciolli V Vaucheret H Ding SW The 21-Nucleotide, but Not 22-Nucleotide, Viral Secondary Small Interfering RNAs Direct Potent Antiviral Defense by Two Cooperative Argonautes in *Arabidopsis thaliana* Plant Cell 2011 1 14 http://www.ncbi.nlm.nih.gov/pmc/articles/PMC3101545/Advance Online Publication 10.1105/tpc.110.082305 PMC3101545 21467580

[B44] Chapman EJ Carrington JC Specialization and evolution of endogenous small RNA pathways Nature Reviews Genetics 2007 8 11 884 896 10.1038/nrg2179 17943195

[B45] Martinez G Donaire L Llave C Pallas V Gomez G High-throughput sequencing of Hop stunt viroid-derived small RNAs from cucumber leaves and phloem Molecular Plant Pathology 2010 11 3 347 359 10.1111/j.1364-3703.2009.00608.x 20447283 PMC6640512

[B46] Vargason JM Szittya G Burgyan J Tanaka Hall TM Size selective recognition of siRNA by an RNA silencing suppressor Cell 2003 115 7 799 811 10.1016/S0092-8674(03)00984-X 14697199

[B47] Xie Z Kasschau KD Carrington JC Negative feedback regulation of Dicer-Like1 in Arabidopsis by microRNA-guided mRNA degradation Current Biology 2003 13 9 784 789 10.1016/S0960-9822(03)00281-1 12725739

[B48] Qu F Ye X Morris TJ Arabidopsis DRB4, AGO1, AGO7, and RDR6 participate in a DCL4-initiated antiviral RNA silencing pathway negatively regulated by DCL1 Proc Natl Acad Sci USA 2008 105 38 14732 14737 10.1073/pnas.0805760105 18799732 PMC2567185

[B49] Shivaprasad PV Rajeswaran R Blevins T Schoelz J Meins F Jr Hohn T Pooggin MM The CaMV transactivator/viroplasmin interferes with RDR6-dependent trans-acting and secondary siRNA pathways in Arabidopsis Nucleic Acids Research 2008 36 18 5896 5909 10.1093/nar/gkn590 18801846 PMC2566869

[B50] Dunoyer P Himber C Voinnet O DICER-LIKE 4 is required for RNA interference and produces the 21-nucleotide small interfering RNA component of the plant cell-to-cell silencing signal Nature Genetics 2005 37 12 1356 1360 10.1038/ng1675 16273107

[B51] Silva TF Correa RL Castilho Y Silvie P Belot JL Vaslin MFS Widespread distribution and a new recombinant species of Brazilian virus associated with cotton blue disease Virology Journal 2008 5 123 1 13 18937850 10.1186/1743-422X-5-123 PMC2583970

[B52] Varkonyi-Gasic E Hellens RP KovaIchuk I, Zemp F qRT-PCR of Small RNAs Plant Epigenetics: Methods and Protocols, Methods in Molecular Biology 2010 631 109 122 20204872 10.1007/978-1-60761-646-7_10

[B53] Artico S Nardeli SM Brilhante O Grossi-de-Sa MF Alves-Ferreira M Identification and evaluation of new reference genes in Gossypium hirsutum for accurate normalization of real-time quantitative RT-PCR data BMC Plant Biology 2010 10 49 2 12 20302670 10.1186/1471-2229-10-49 PMC2923523

[B54] Zhao S Fernald RD Comprehensive algorithm for quantitative real -time polymerase chain reaction Journal of Computational Biology 2005 12 1047 1064 10.1089/cmb.2005.12.1047 16241897 PMC2716216

[B55] Letunic I Doerks T Bork P SMART 6: recent updates and new developments Nucleic Acids Research 2009 37 D229 D232 10.1093/nar/gkn808 18978020 PMC2686533

[B56] Vandesompele J De Preter K Pattyn F Poppe B Van Roy N De Paepe A Speleman F Accurate normalization of real-time quantitative RT-PCR data by geometric averaging of multiple internal control genes Genome Biology 2002 3 7 1 12 12184808 10.1186/gb-2002-3-7-research0034 PMC126239

