# Supplementary figures and images for: Profile of small interfering RNAs from cotton plants infected with the polerovirus Cotton leafroll dwarf virus
Source: BMC Mol Biol. 2011 Aug 24;12:40. doi: 10.1186/1471-2199-12-40 (PMC3189115; doi:10.1186/1471-2199-12-40)

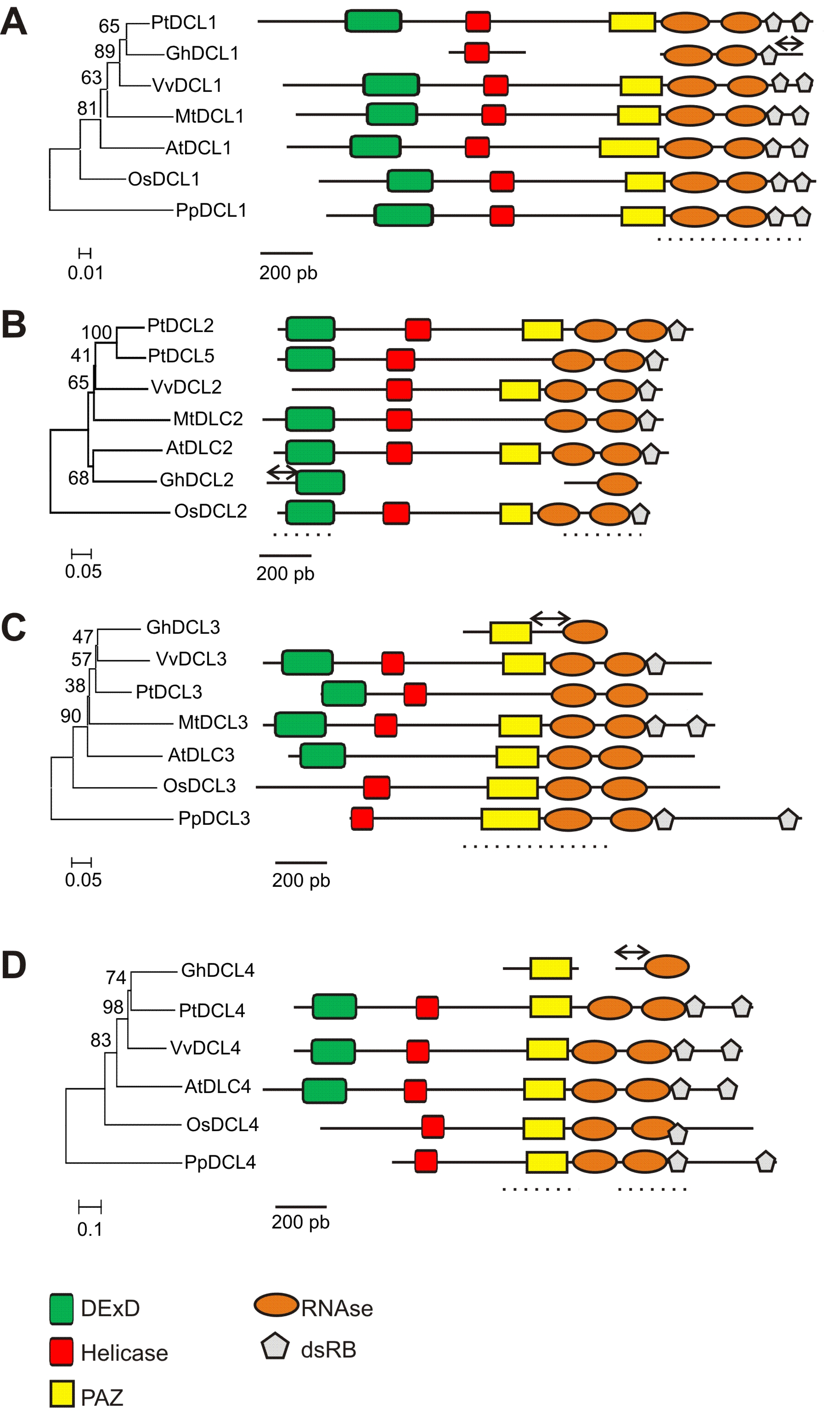

Supplement: Additional file 1 — Phylogenetic relationship between cotton Dicer ribonucleases and their homologues in other species. A, B, C, and D, Unrooted Neighbor-joining tree constructed with DCL1, DCL2, DCL3, or DCL4 homologue sequences, respectively. Species used in the phylogeny were as follows: Arabidopsis thaliana (At), Gossypium hirsutum (Gh), Medicago truncatula (Mt), Oryza sativa (Os), Physcomitrella patens (Pp), Populus trichocarpa (Pt) and Vitis vinifera (Vv). Dashes below each tree represent amino acid regions used in alignment. Arrows represent fragments analyzed by qPCR. Bootstrap values from 1,000 replicates were used to assess the robustness of the trees. All DCL sequences, except cotton DCLs, were downloaded from Phytozome 6.0 http://www.phytozome.net/. ESTs from G. hirsutum containing incomplete DCL sequences were obtained from the NCBI database. The GhDCL1 consensus sequence was constructed with ESTs DT564382.1 (Helicase domain), and ES804646.1, together with DW238156.1 (two RNAse III and one Double stranded RNA binding (dsRB) domain). The GhDCL2 consensus sequence was constructed from two ESTs: DW484144 (DEAD-like helicases superfamily (DExD) domain) and ES806737 (second RNAse III domain). The GhDCL3 sequence was constructed from the ESTs DW477937 and DR462994 (PAZ and RNAse III domains, respectively). The GhDCL4 consensus sequence was constructed with ESTs ES841096 (PAZ domain) and DT568872 (RNAse III domain). Smart database [55] was used to identity DCL domains from their amino acid sequences. [file 1471-2199-12-40-S1.JPEG]

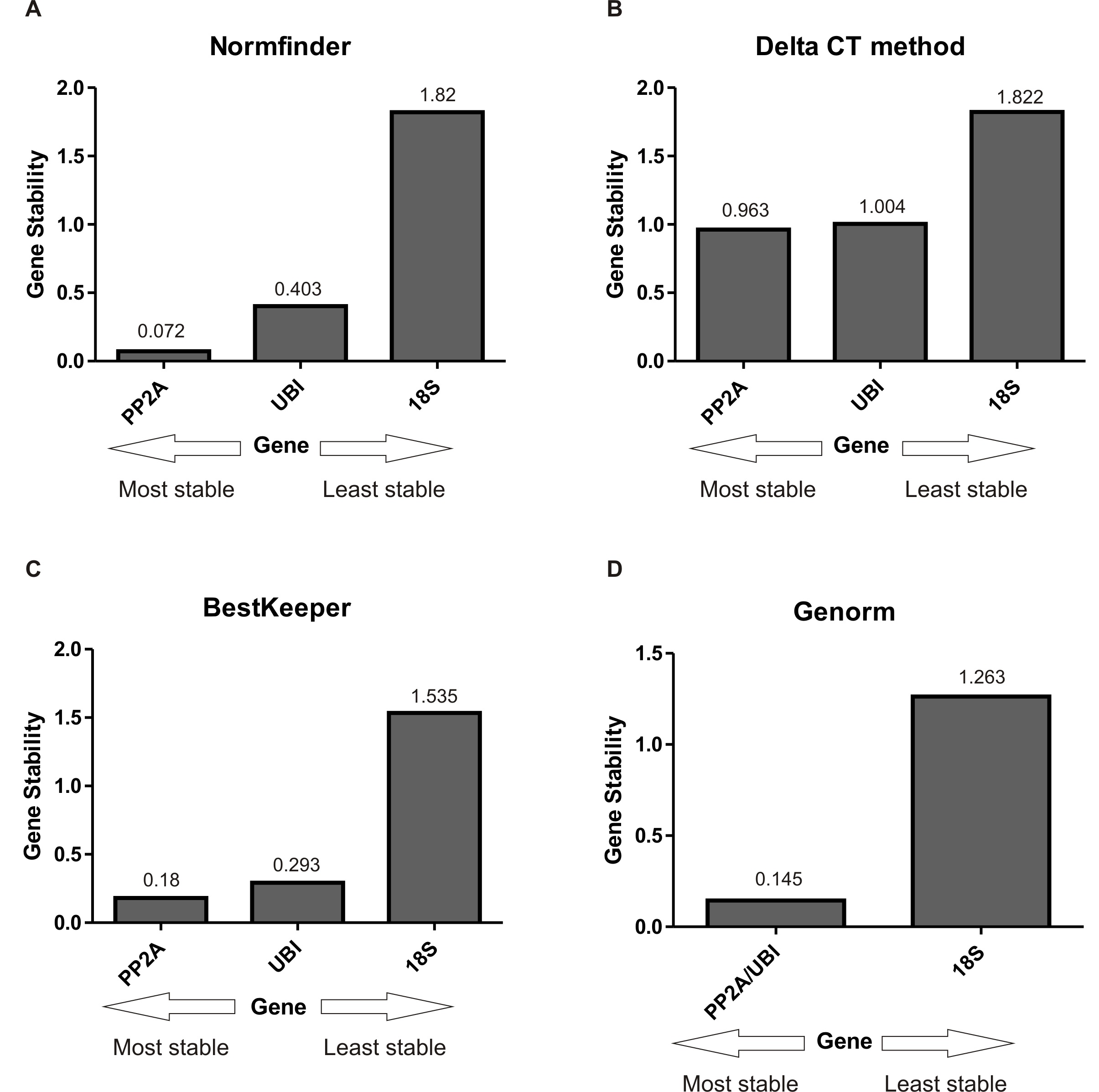

Supplement: Additional file 5 — Determination of reference genes for use in these experimental conditions. Expression stability values of polyubiquitin (UBI), the catalytic subunit of phosphatase 2A (PP2A), and 18S ribosomal RNA (18S) candidate reference genes obtained by different algorithms. (A) Normfinder. (B) Delta CT method. (C) BestKeeper. (D) Genenorm. In Gennorm analysis, 0.15 is the cut-off value below which the inclusion of an additional reference gene is not required [56]. All analyses were performed via the Cotton EST Database http://www.leonxie.com/index.php. [file 1471-2199-12-40-S5.JPEG]

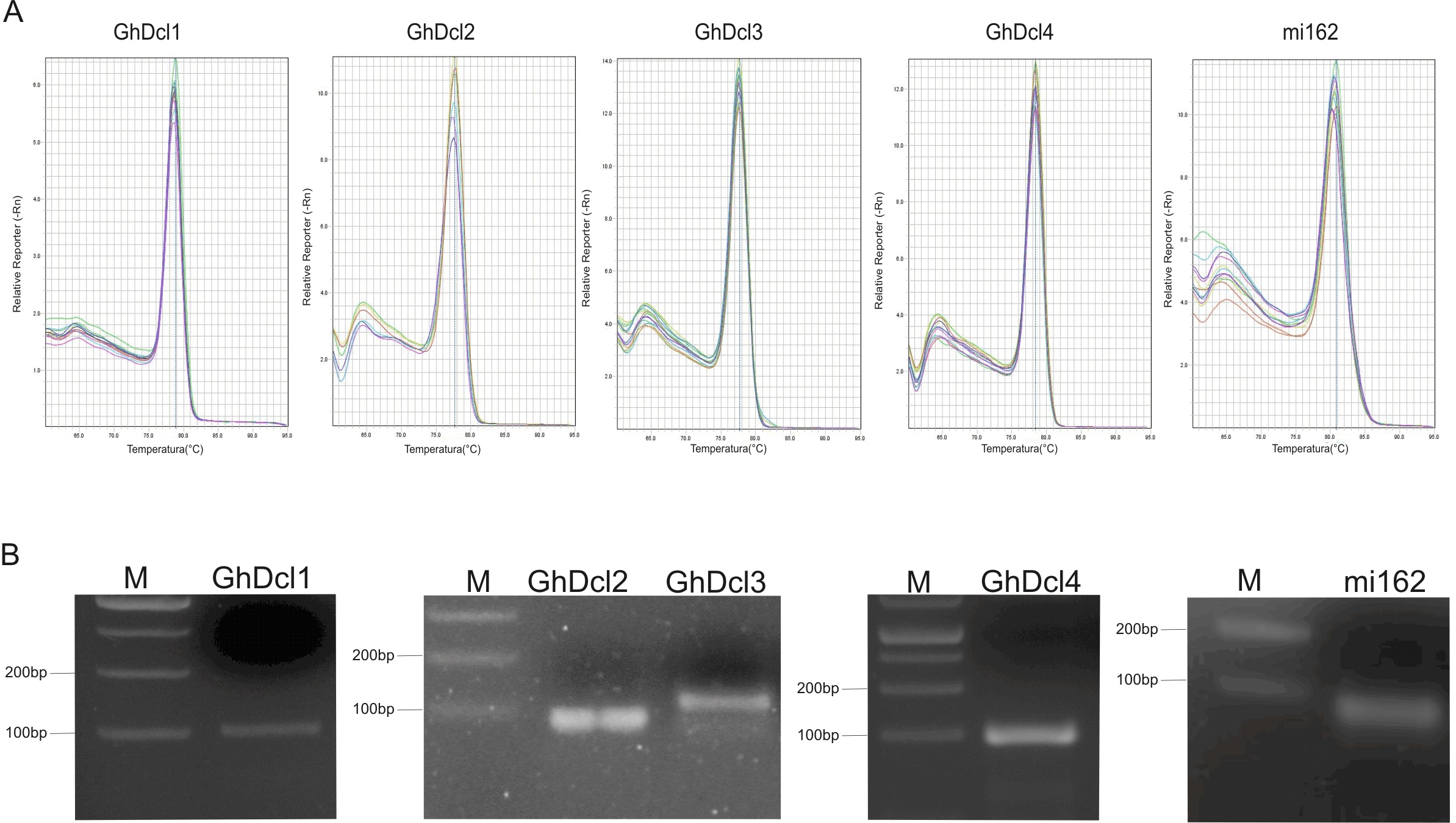

Supplement: Additional file 6 — Test of specificity of RT-qPCR primers. (A) Melting curves of the four GhDcls and Gh-miR162 sequence-related RNAs after RT-qPCR using SYBR-green. (B) Non-denaturing agarose (2.0%) gel electrophoresis showing amplification of single products with the expected size for each of the GhDCL gene transcripts and Gh-miR162. M represents O'GeneRuler 100 bp DNA Ladder (Fermentas). [file 1471-2199-12-40-S6.JPEG]
